# Investigation of Iron(III) Tetraphenylporphyrin as a Redox Flow Battery Anolyte: Unexpected Side Reactivity with the Electrolyte

**DOI:** 10.1021/acs.jpcc.3c01763

**Published:** 2023-06-01

**Authors:** Nathan H. Mitchell, Noémie Elgrishi

**Affiliations:** Louisiana State University, Baton Rouge, Louisiana 70803, USA

## Abstract

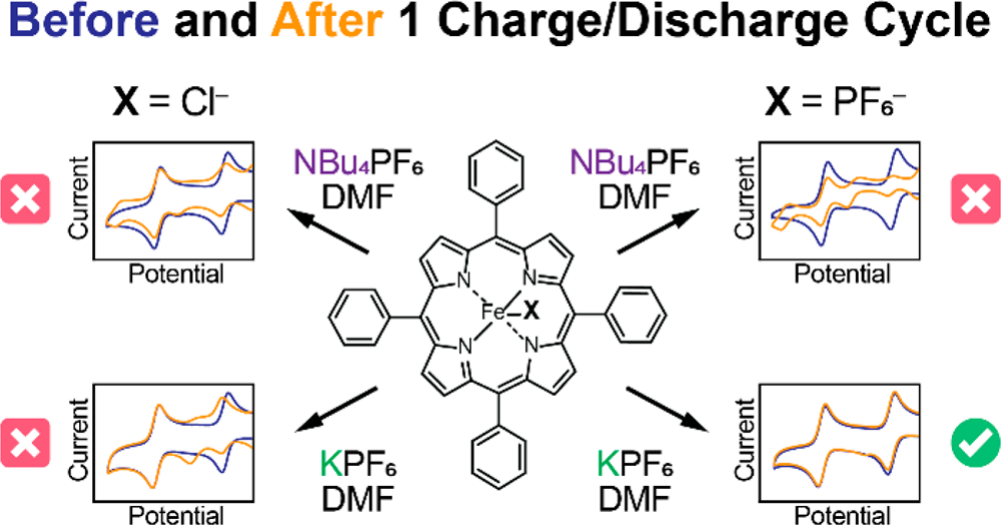

Redox flow batteries (RFBs) present an opportunity to bridge the gap between the intermittent availability of green energy sources and the need for on-demand grid level energy storage. While aqueous vanadium-based redox flow batteries have been commercialized, they are limited by the constraints of using water as an electrochemical solvent. Nonaqueous redox flow battery systems can be used to produce high voltage batteries due to the larger electrochemical window in nonaqueous solvents and the ability to tune the redox properties of active materials through functionalization. Iron porphyrins, a class of organometallic macrocycles, have been the subject of many studies for their photocatalytic and electrocatalytic properties in nonaqueous solvents. Often, iron porphyrins can undergo multiple redox events making them interesting candidates for use as anolytes in asymmetrical redox flow batteries or as both catholyte and anolyte in symmetrical redox flow battery systems. Here the electrochemical properties of Fe(III)TPP species relevant to redox flow battery electrolytes are investigated including solubility, electrochemical properties, and charge/discharge cycling. Commonly used support electrolyte salts can have reactivities that are often overlooked beyond their conductivity properties in nonaqueous solvents. Parasitic reactions with the cations of common support electrolytes are highlighted herein, which underscore the careful balance required to fully assess the potential of novel RFB electrolytes.

## Introduction

The recent shift away from fossil fuels and toward alternative energy sources has led to an increased focus on energy storage systems.^1^ Redox flow batteries (RFBs) store energy in the form of oxidized and reduced redox couples in physically separated solutions allowing long-term storage and facile scalability.^2−4^ The most well-developed RFB chemistry consists of vanadium salts in acidic solutions, which limit the cell potential to the electrochemical window of water.^5−7^ The use of a nonaqueous RFB permits a greater cell potential and greater synthetic control. This has led to RFBs that use organic^8−10^ and organometallic^11−13^ electrolytes with high cell potentials,^14^ multiple electron transfers per molecule,^15,16^ or both.^17,18^

**Scheme 1:**
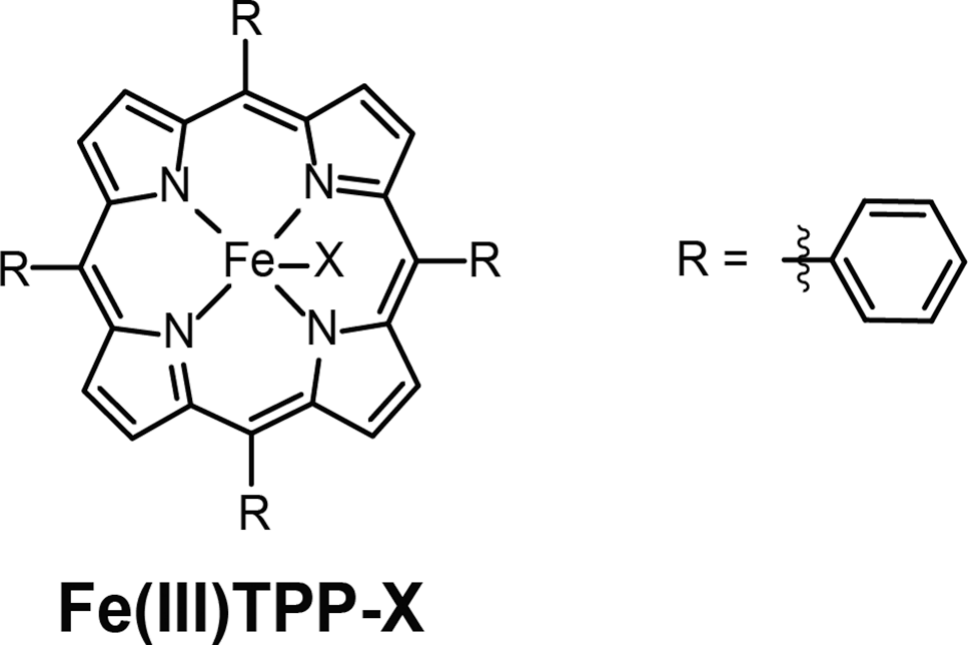
Porphyrins under Study X = Cl^–^ for Fe-TPP-Cl or X = outer-sphere PF_6_^–^ for Fe-TPP-PF_6_.

Iron porphyrins belong to a large class of organometallic compounds which share a heme macrocycle core and exhibit multiple electron transfers over a wide potential range in nonaqueous systems.^19,20^ Various functionalized iron porphyrins have been studied as chemical, photo-, and electrocatalysts for reactions including CO_2_ reduction,^21−26^ reduction of fluranes,^27^ O_2_ reduction,^28−30^ proton reduction,^30,31^ and cycloaddition^32,33^ among others.^30,34^ In regard to CO_2_ reduction electrocatalysis, iron tetraphenylporphyrins (Fe-TPP, Scheme 1) typically undergo three successive one-electron transfers to form the catalytically active formally Fe(0) tetraphenylporphyrin.^26,35^ The emphasized electrochemical stability of these systems was a driver to explore the potential use of Fe-TPP to store charge in nonaqueous RFBs. Indeed, the porphyrin macrocycle allows for a high degree of molecular tuning by functionalization of the phenyl group; examples include addition of highly electron withdrawing fluoro groups, charged groups, and protonated groups,^36−39^ which can have significant effects on the redox chemistry of the complex and its electrocatalytic activity.^20,28,40^ These qualities make iron tetraphenylporphyrins an appealing platform for examining the charge/discharge cycling stability of a group of compounds that have been extensively described in an electrocatalytic setting.

A less addressed factor of redox flow battery systems is the choice of support electrolyte. Supporting electrolyte anions are usually hexafluorophosphate, tetrafluoroborate, or perchlorates, although perchlorates are avoided when possible for safety considerations.^1^ Tetraalkylammonium salts are some of the most commonly used cations in support electrolytes in nonaqueous systems due to their resistance to reduction and relatively high molar conductivity in organic solvents.^41−45^ The reactivity of the ions that make up support electrolytes is often ignored over the large potential windows they enable but should not be overlooked. Hexafluorophosphate salts with Lewis acidic cations, such as NaPF_6_ and LiPF_6_, have been known to hydrolyze in the presence of trace water to form HF.^46,47^ Alternatively, tetraalkylammonium salts are known to form ion pairs with reduced species that can affect thermal and photochemical processes.^48^ When paired with halides, they are also known to undergo nucleophilic reactions to form neutral trialkylamines as well as alkenes and alkanes through radical and disproportionation reactions.^49^ Reports with alternative support electrolytes in RFB studies such as alkali metal salts^50^ or more recently zinc(II) perchlorate^13^ are occasionally used but usually to achieve a specific goal, such as reduced cost or in the example with zinc(II) perchlorate to catalyze a concerted two-electron reaction.^13^ Overall, there is a lack of reports addressing the specific challenges posed by supporting electrolyte degradation reactions. This is of critical importance as redox active electrolytes assessed for RFB charge storing may be overlooked or be perceived as nonsuitable due to unrecognized electrolyte-induced degradations.

In this study, we examine the electrochemical behavior of iron porphyrins relevant to RFB chemistry and demonstrate the issues encountered when adapting a well-studied electrocatalyst for use as an RFB electrolyte. The simplest iron tetraphenylporphyrin, commercially available iron(III) tetraphenylporphyrin chloride (Fe(III)TPP-Cl), was chosen as a model iron porphyrin as it is thoroughly characterized in nonaqueous media. We report the presence of parasitic reactions during charge/discharge cycling, and investigate the role counterions and support electrolyte play in promoting or mitigating charge/discharge cycling stability.

## Materials and Methods

### General Considerations

UV–vis absorption spectra were collected using an Ocean Optics UV–vis spectrometer with a DH-2000 BAL dual source (deuterium and tungsten) and OCEAN-FX-XRI-ES diode array detector. Electrochemical measurements were recorded on either a Biologic SP 300 or Biologic BP 300 potentiostat. FTIR spectra were collected using a Bruker Alpha FT-IR spectrometer with a Pt-diamond single-bounce ATR cell and a DTGS detector.

### Materials

The following chemicals were used as received without further purification: Fe(III)TPP-Cl (Beantown Chemicals, ≥97.0%), ferrocene (Alfa Aesar, 99%), and silver hexafluorophosphate (Strem Chemicals, ≥99%). Tetrabutylammonium hexafluorophosphate (NBu_4_PF_6_) was recrystallized from hot ethanol and dried at 80 °C under vacuum. Both dichloromethane (BDH Chemicals, ≥99.5%) and *N*,*N*-dimethylformamide (ACS grade, ≥99.8%) were dried over 3 Å activated molecular sieves (10% w/v%) for no less than 2 days before using. Potassium hexafluorophosphate (Beantown Chemical, ≥99%) solutions in DMF were prepared and dried over 3 Å activated molecular sieves (10% w/v%) before use.

### Synthesis

Fe(III)TPP-PF_6_ was synthesized following literature procedures.^32,34^ Briefly, Fe(III)TPP-Cl (100.5 mg, 0.116 mmol) and AgPF_6_ (38.2 mg, 0.116 mmol) were combined in a flask with dichloromethane (12 mL) and stirred overnight at room temperature, shielded from light. The solution was then filtered before being concentrated to dryness to yield a purple solid. The solid was dried overnight under vacuum at 40 °C. Product identity was confirmed by FTIR by the disappearance of the 381 cm^–1^ stretching frequency corresponding to Fe–Cl upon exchange of the Cl^–^ counterion for PF_6_^–^.^34^

### Solubility

Solubilities were determined from UV–vis absorbance spectroscopy measurements following reported procedures.^51^ Briefly, calibration curves were prepared with known concentrations of each porphyrin at low concentrations. A saturated solution of each porphyrin was then diluted into the range of the calibration curve. The absorbance of the diluted sample was then used to calculate the concentration of the saturated solution using the soret band of Fe(III)TPP.

### Electrochemical Measurements

Cyclic voltammograms (CVs) were collected using a three-electrode setup with a glassy carbon working electrode (3 mm diameter, CH instruments), platinum counter electrode (2 mm diameter, CH instruments), and a silver wire pseudo-reference-electrode stored in 0.25 M NBu_4_PF_6_ in anhydrous solvent and separated from the electrochemical cell by a glass frit. The electrochemical cell was composed of a 20 mL borosilicate glass scintillation vial with a custom-made PTFE cap. Working electrodes were polished using type N Alpha alumina powder (0.05 μm, Electron Microscopy Sciences) on a microcloth polishing pad (CH instruments) and then pretreated by cycling between upper and lower potential boundaries of the electrochemical window for the experiment in a solution containing only the electrolyte. Measurements were taken in a dried and degassed DMF solution with 0.1 M recrystallized NBu_4_PF_6_ or 0.1 M KPF_6_. Iron porphyrins were added to 5 mL of electrolyte solution to give the desired concentration. Cyclic voltammograms of ferrocene were recorded at the beginning and end of each experiment to check for possible reference electrode drift, and potentials were referenced to the *E*_1/2_ value for the Fc/Fc^+^ couple. Solutions were sparged with N_2_ before each measurement.

### H-Cell Charge/Discharge Cycling

Charge/discharge cycling was performed in a custom glass h-cell with a size P5 ultrafine frit separating the working and counter electrode compartments (Figure S1) using a three-electrode setup with a reticulated vitreous carbon (RVC) working electrode (4.5 × 6 mm, Duocel 100 ppi), RVC counter electrode (4.5 × 6 mm, Duocel 100 ppi), and a silver wire pseudoreference electrode stored in 0.25 M NBu_4_PF_6_ in anhydrous solvent and separated from the electrochemical cell by a Vycor glass frit (Gamry Instruments). The electrolyte solution contained the porphyrin and 0.1 M support electrolyte. The working electrode compartment contained 4 mL of electrolyte solution, and the counter electrode compartment contained 4 mL of ferrocene solution at 4 equiv. Both compartments were stirred constantly at 1500 rpm throughout the experiment. Currents were held at ±1.0 mA until overpotentials of 0.30 V were reached for the most positive and most negative oxidations and reductions. Unless otherwise noted, the charge cycle lasted until 100% state of charge (SOC) was reached or after 20 min at the potential limit.

### Spectro-electrochemistry

Spectro-electrochemical measurements were made using a Honeycomb Platinum electrode (Pine Research) with a silver wire pseudoreference electrode stored in 0.25 M NBu_4_PF_6_ in anhydrous CH_3_CN and separated from the electrochemical cell by a glass frit. The cuvette was a thin-layer quartz cuvette cell (Pine Research, 1.7 mm path length). UV–vis absorbance spectra were recorded, while the potential was varied. The spectro-electrochemical cell was sparged with N_2_ for no less than 20 min before electrochemical measurements were collected, and the solution was blanketed with N_2_ throughout.

## Results and Discussion

### Solubility

The solubility of Fe(III)TPP-Cl was found to be 5.2 mM in neat DMF by UV–vis absorbance spectroscopy (Figure S2). The solubility of Fe(III)TPP was increased to 23.7 mM by exchanging the chloride counterion for hexafluorophosphate (Figure S3). While the low solubility of Fe(III)TPP-Cl is a concern, the Fe(III)TPP-Cl framework allows for various ligand modifications, which can increase solubility. Reports show that the solubility of M-TPP systems can be enhanced through a variety of ligand modifications, for example functionalization of the phenyl rings with n-octyl triazole chains,^52^ with triphenylbenzene rings,^53^ or with ether linkers.^54^ Such modifications have been reported to increase solubility to 50 mM or greater, which would allow an electron equivalent molarity of 150 mM if all three redox events are retained. Alternatively, H_2_TPP has been paired with Ketjen Black (KB) to increase the functional solubility of H_2_TPP in a symmetric redox flow battery that utilized a suspension electrolyte, wherein the conductive KB allowed electron transport into aggregated H_2_TPP.^55^

### Electrochemical Properties

**Scheme 2:**
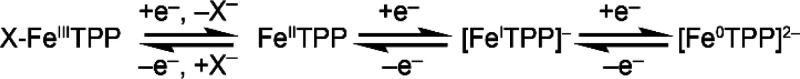
Expected Electron Transfers to Fe(III)TPP-X

Based on previous reports, the Fe-TPP porphyrins are expected to undergo three consecutive one-electron transfers to make the formal Fe(0) species^35^ following the general transformations in Scheme 2:^56^

**Figure 1:**
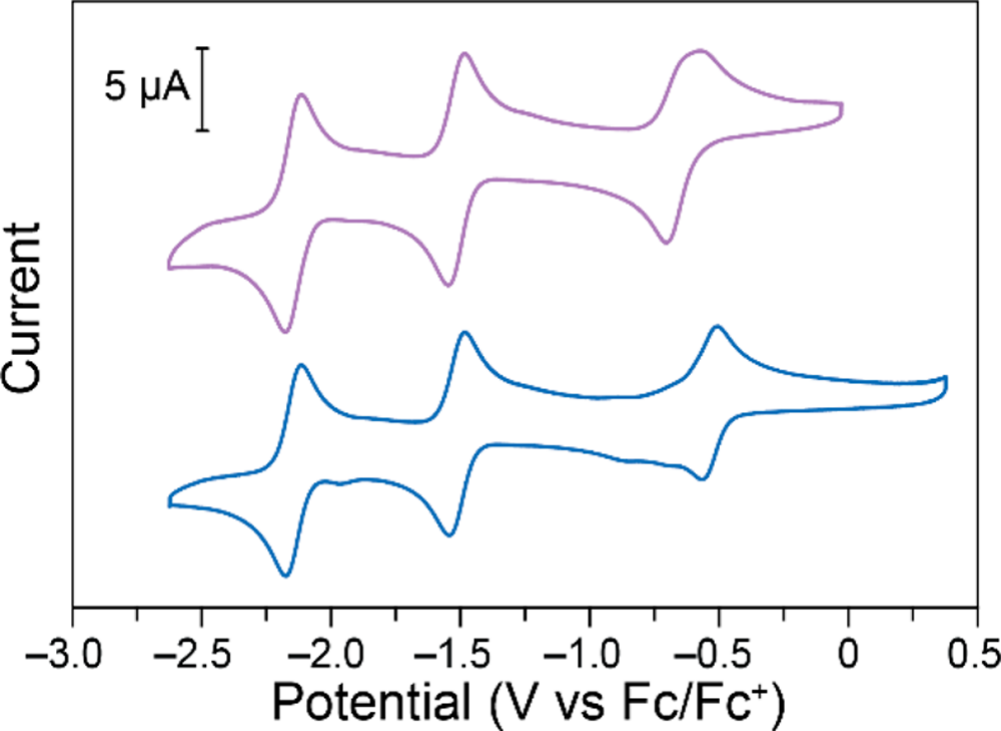
Cyclic voltammograms on glassy carbon electrodes of 0.59 mM Fe(III)TPP-Cl (top) and 0.54 mM Fe(III)TPP-PF_6_ (bottom) in 0.1 M NBu_4_PF_6_ in DMF. Data recorded at 100 mV s^–1^.

As expected, three redox couples were observed for Fe-TPP-Cl in DMF with 0.1 M NBu_4_PF_6_ (Figure 1, top trace). Half-wave potentials were determined for each couple by averaging the anodic and cathodic peak potentials and are shown in Table 1. Utilizing these three redox events, Fe(III)TPP-Cl has a theoretical open circuit potential of 1.51 V were it to be deployed in a symmetric redox flow battery system as both catholyte and anolyte. The voltammograms of FeTPP-Cl are stable on the time scale of cyclic voltammetry experiments in NBu_4_PF_6_ in DMF. This was determined by the stable overlapping traces observed when multiple cycles were collected (Figure S4). Variable scan rate studies (Figure S5) determined each redox event to be diffusion-controlled on the CV time scale from the linear relationship between peak currents and the square root of the scan rate. This allowed the calculation of diffusion coefficients (*D*_0_) using the Randles-Sevcik equation (Figure S6), which ranged from 2.6 × 10^–6^ to 3.2 × 10^–6^ cm^2^ s^–1^ (Table 1).

**Table 1:** Electrochemical Properties in NBu_4_PF_6_ in DMF^a^

		Fe(III/II)	Fe(II/I)	Fe(I/0)
FeTPP-Cl	*E*_1/2_^b^	–0.638	–1.515	–2.146
	Δ*E*_p_^c^	133 mV	65 mV	65 mV
	*D*_0_ (cm^2^ s^–1^)	2.6 × 10^–6^	2.7 × 10^–6^	3.2 × 10^–6^
	*k*_0_ (cm s^–1^)	0.16 × 10^–2^	4.2 × 10^–2^	3.0 × 10^–2^
FeTPP-PF_6_	*E*_1/2_^b^	–0.533	–1.511	–2.141
	Δ*E*_p_^c^	64 mV	60 mV	62 mV
	*D*_0_ (cm^2^ s^–1^)	1.5 × 10^–6^	3.3 × 10^–6^	4.3 × 10^–6^
	*k*_0_ (cm s^–1^)	3.8 × 10^–2^	6.1 × 10^–2^	5.7 × 10^–2^

a Data collected in 0.1 M NBu_4_PF_6_ in DMF on glassy carbon working electrodes.

b Potentials given in V vs Fc/Fc^+^.

c Δ*E*_p_ values for CVs collected at 100 mV s^–1^.

The Nicholson method (Figure S7) was used to determine heterogeneous electron transfer rate constants (*k*_0_) for Fe(III)TPP-Cl. These were found to range from 1.6 × 10^–3^ to 4.2 × 10^–2^ cm s^–1^ (Table 1). The values for both diffusion coefficients and rate constants are comparable to those seen in aqueous vanadium RFBs and proposed nonaqueous RFB systems.^3,13,44,57^

In DMF with 0.1 M NBu_4_PF_6_, the oxidation of Fe(II) to Fe(III) for FeTPP-Cl appears as a broad peak with a 133 mV peak-to-peak separation. This is a larger peak-to-peak separation than would be expected for a fully reversible system. This is likely due to the exchange of the chloride counterion required during the Fe(III)/Fe(II) redox event (Scheme 2).^56,58^ To test if reversibility could be enhanced with addition of a halide, CVs were collected in the presence of 20 equiv of KBr as a soluble halide source in DMF. The reversibility of the Fe(III)/Fe(II) wave was improved slightly, with peak-to-peak separation decreasing to 104 mV (Figure S8).

In an attempt to more effectively increase reversibility of the Fe(III)/Fe(II) redox couple, the Cl^–^ counterion was replaced with PF_6_^–^, matching the supporting electrolyte. Fe(III)TPP-PF_6_ was synthesized by reacting Fe(III)TPP-Cl with AgPF_6_ in DCM.^32^ The electrode reactions of Fe(III)TPP-PF_6_ in NBu_4_PF_6_ in DMF are largely unchanged compared to Fe(III)TPP-Cl except for the Fe(III)/Fe(II) redox couple, which is shifted positively by 100 mV (Figure 1, bottom trace). This would give an open circuit potential of 1.61 V if operated in a symmetric RFB system. Scan rate variation cyclic voltammetry studies also showed that the Fe(III)/Fe(II) redox couple is more reversible with the more weakly coordinating PF_6_^–^ counterion (Figures S9–12). As with the Cl^–^ complex, Fe(III)TPP-PF_6_ is stable on the CV time scale (Figure S9). The heterogeneous electron transfer rate constant for the Fe(III)/Fe(II) redox couple was improved as well, while the diffusion coefficients and the remaining rate constants were virtually unchanged as expected (Table 1).

### H-Cell Charge/Discharge Cycling

**Figure 2:**
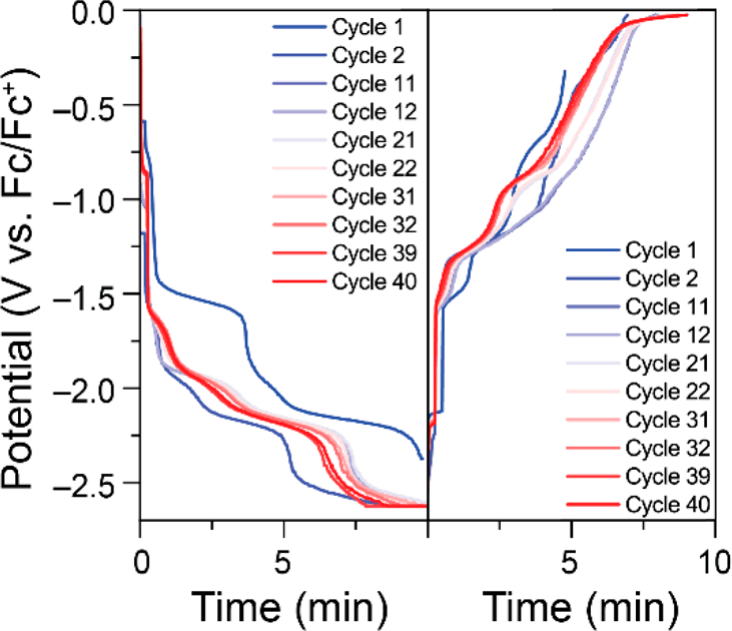
Selected representative potential vs time curves for 0.48 mM Fe(III)TPP-Cl in 0.1 M NBu_4_PF_6_ in DMF during charge (left) and discharge (right) cycling.

**Figure 3:**
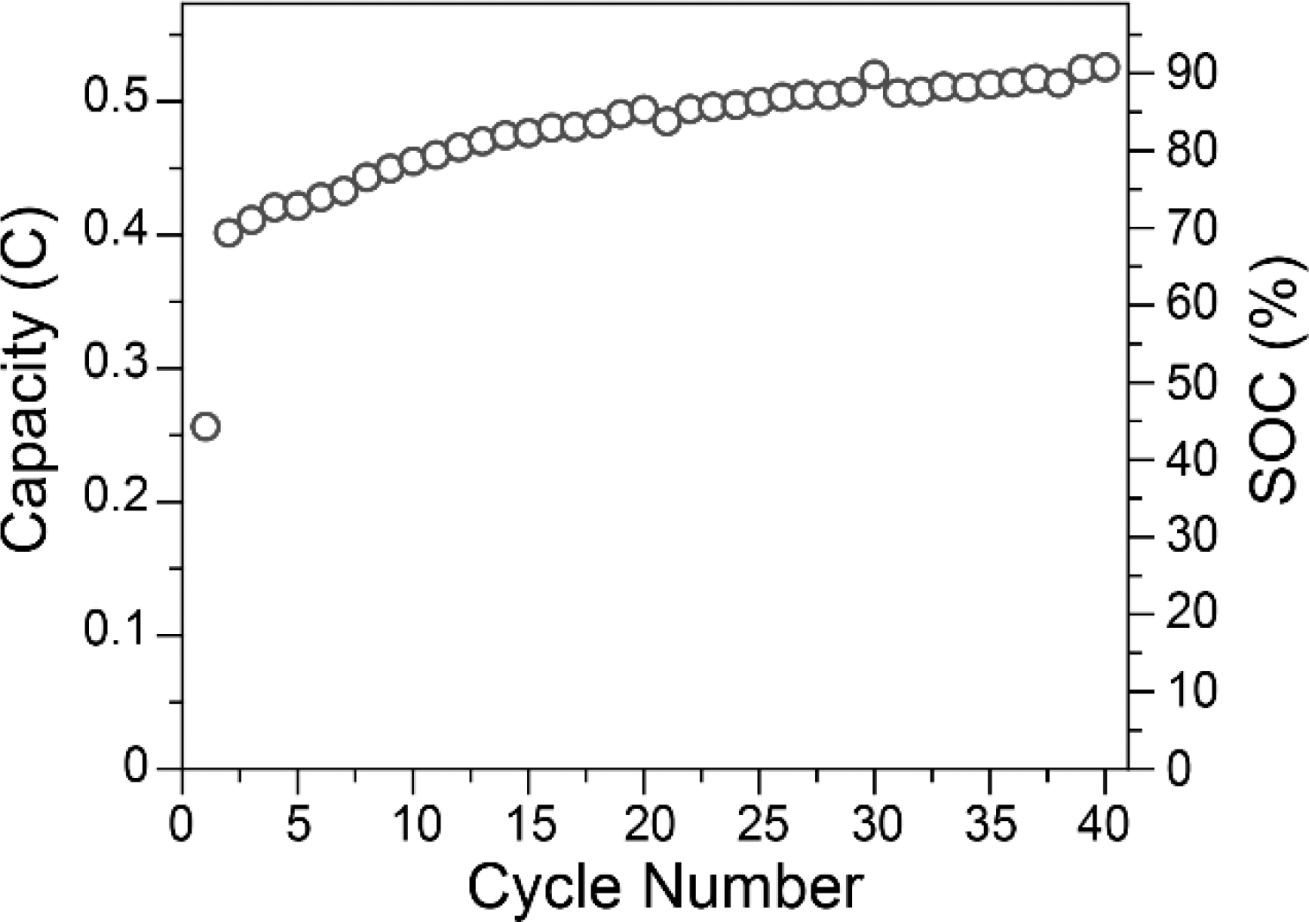
Discharge capacities and % SOC for charge/discharge cycling of 0.48 mM Fe(III)TPP-Cl in 0.1 M NBu_4_PF_6_ in DMF.

Charge/discharge cycling was performed using galvanostatic charging with potential limitations (GCPL) wherein a current of ±1.0 mA was used for charging/discharging in 0.1 M NBu_4_PF_6_ in DMF. The working electrode compartment was filled with ca. 0.5 mM porphyrin as anolyte, and the counter electrode compartment was filled with 4 equiv of ferrocene to act as catholyte. During charge, a cathodic current was applied until an overpotential of 0.3 V for the reduction of Fe(I) to Fe(0) was reached. During discharge, an anodic current was applied until an overpotential of 0.3 V for the oxidation of Fe(II) to Fe(III) was reached (Figure 2). To ensure complete accurate charging, the charge cycle was held at the limiting potential for 20 min or until 100% SOC was attained. Thus, capacities are based solely on discharge cycling (Figure 3).

**Figure 4:**
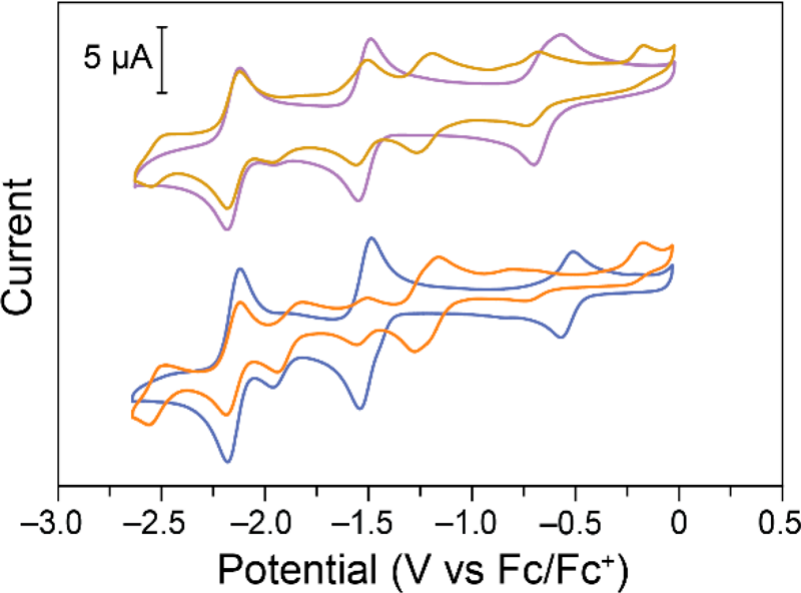
Cyclic voltammograms of Fe(III)TPP-X collected on glassy carbon electrodes in 0.1 M NBu_4_PF_6_ in DMF at 100 mV s^–1^ before and after a single charge/discharge cycle. Top: 0.48 mM Fe(III)TPP-Cl before (purple) and after (yellow). Bottom: 0.52 mM Fe(III)TPP-PF_6_ before (blue) and after (orange).

The difference in capacities between the first charge and first discharge cycle are likely due to cell conditioning.^59,60^ During the first 40 charge/discharge cycles, the discharge capacity gradually increased until it reached 90% of theoretical capacity. This was unexpected and likely an indication of unwanted side reactions or possibly crossover.^61^ Further charge/discharge cycles of Fe(III)TPP-Cl (Figures S13 and S14) resulted in an apparent plateau of discharge capacity through 57 cycles. However, CVs collected after the 1st cycle (Figure 4, top traces) and 57th cycle (Figure S15) showed significant degradation of Fe(III)TPP-Cl. As can be seen in the yellow CV trace in the top part of Figure 4, after a single charge/discharge cycle, all three redox couples are affected. Peak currents for the oxidations and reductions of each couple are diminished, and the Fe(III)/Fe(II) couple is shifted negative. Simultaneously, two new redox couples and a new oxidation are formed. At the end of 57 charge/discharge cycles, only the Fe(I)/Fe(0) couple remains, while the preceding redox couples are no longer apparent (Figure S15). After the 57th cycle, charge/discharge cycling was performed without holding the charge capacity to 100% state of charge. This resulted in a swift and constant drop of discharge capacity of 0.6% per cycle (Figure S14), and CVs collected after 151 cycles resulted in the almost complete degradation of Fe(III)TPP-Cl (Figure S15). This indicates that the discharge capacity recorded was not due to charge/discharge cycling of Fe(III)TPP-Cl but due to other parasitic reactions and possibly some crossover of the Fc catholyte through the porous glass separator. While the type of frit used (P5 ultrafine) is frequently used to specifically overcome any possible crossover issues,^16,62−65^ some studies have reported some crossover with these materials.^16,62,65^

Since Fe(III)TPP-PF_6_ was shown to have more desirable electrochemical properties than Fe(III)TPP-Cl, a single charge/discharge cycle was performed to determine if similar degradation reactions would occur. CVs collected before and after a charge/discharge cycle (Figure 4, bottom) as well as the potential vs time curves (Figure S16) show that a similar type of degradation indeed occurred.

Despite the widespread use of Fe porphyrins as electrocatalysts in NBu_4_PF_6_ electrolyte in DMF, the results above are explained by the underappreciated reaction between the generated [Fe(0)TPP]^2–^ and the NBu_4_^+^ support cations.^66,67^ This Fe(0) species is expected to be generated when starting from both Fe(III)TPP-Cl and Fe(III)TPP-PF_6_, which explains the similar degradations observed after a charge/discharge cycle.

The redox couple at ca. −1.23 V vs Fc/Fc^+^ is assigned to the quasireversible redox reaction of an alkyl iron(II) complex, while the oxidation at ca. −0.17 V vs Fc/Fc^+^ can be assigned to the oxidation of an electrogenerated alkyl iron(III).^66,67^ While the small redox couple negative of the Fe(I)/Fe(0) redox couple has not been formally assigned, it is expected to be a result of the degradation reactions.

It should be emphasized that the reaction of [Fe(0)TPP]^2–^ with NBu_4_^+^ is slower than the time scale of cyclic voltammetry,^66,67^ which means the charge/discharge cycling tests were necessary to highlight its importance. This is critical and underappreciated in electrochemical studies relying solely on CV.

**Figure 5:**
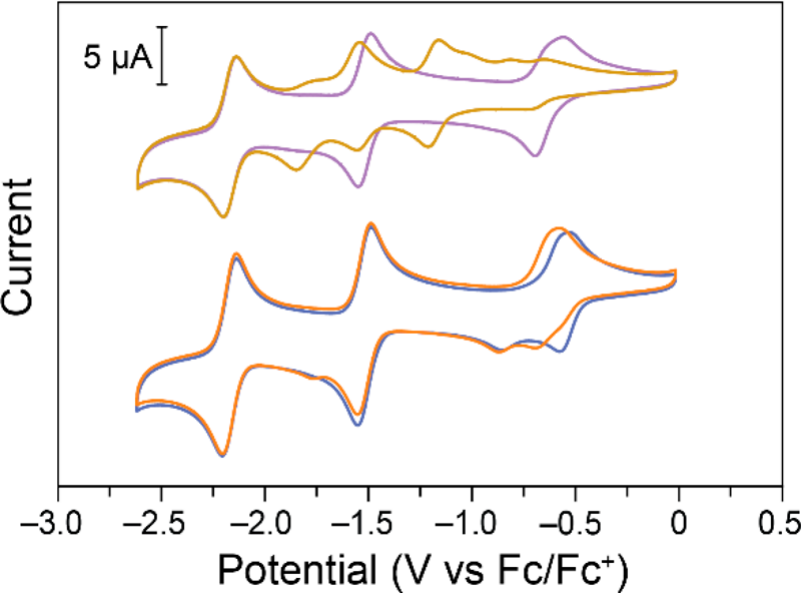
Cyclic voltammograms of Fe(III)TPP-X collected on glassy carbon electrodes in 0.1 M KPF_6_ in DMF at before and after a single charge/discharge. Top: 0.53 mM Fe(III)TPP-Cl before (purple) and after (yellow). Bottom: 0.68 mM Fe(III)TPP-PF_6_ before (blue) and after (orange).

The initial paper by Ryan et al. reporting the reaction between NBu_4_^+^ and [Fe(0)TPP]^2–^ showed that the same degradation did not happen when NaClO_4_ and LiClO_4_ were used as support electrolytes.^66^ Given the undesirable nature of perchlorate salts, KPF_6_ was tested as an alternative. A study by Sanford showed that replacing NBu_4_PF_6_ with KPF_6_ had little effect on the electrochemical properties of a benzoylpyridinium derivative in MeCN and had a minimal effect on cycling stability, which made KPF_6_ a good test candidate.^50^ CVs of Fe(III)TPP-Cl and Fe(III)TPP-PF_6_ showed little difference in KPF_6_ in DMF (Figure 5) compared to NBu_4_PF_6_ in DMF (Figure 1). As in NBu_4_PF_6_, CVs recorded over multiple cycles showed that FeTPP-PF_6_ was stable on the CV time scale in KPF_6_ in DMF (Figure S17).

Scan rate-dependent CVs (Figures S18–S20) were collected for Fe(III)TPP-PF_6_ to determine its electrochemical properties in 0.1 M KPF_6_ in DMF and are summarized in Table 2. The *E*_1/2_ values are effectively unchanged for the Fe(III)/Fe(II) and Fe(II)/Fe(I) redox couples, while the Fe(I)/Fe(0) redox couple is shifted negative by 20 mV. The diffusion coefficients and heterogeneous electron transfer rate constants are all comparable to those found for Fe(III)TPP-PF_6_ in NBu_4_PF_6_ in DMF.

**Table 2:** Electrochemical Properties in KPF_6_ in DMF^a^

		Fe(III/II)	Fe(II/I)	Fe(I/0)
FeTPP-PF_6_	*E*_1/2_^b^	–0.538	–1.513	–2.166
	*D*_0_ (cm^2^ s^–1^)	2.9 × 10^–6^	3.2 × 10^–6^	3.3 × 10^–6^
	*k*_0_ (cm s^–1^)	6.0 × 10^–2^	5.1 × 10^–2^	4.0 × 10^–2^

a Data collected in 0.1 M NBu_4_PF_6_ in DMF on glassy carbon working electrodes.

b Values give in V vs Fc/Fc^+^.

To see if KPF_6_ would be a suitable support electrolyte for charge/discharge cycling of Fe(III)TPP, single charge/discharge cycles were performed on both Fe(III)TPP-PF_6_ and Fe(III)TPP-Cl in 0.1 M KPF_6_ in DMF.

Upon completing a single charge/discharge cycle of FeTPP-Cl in KPF_6_ (Figure S21), the Fe(III)/Fe(II) redox couple shifted negatively, the Fe(II)/Fe(I) redox couple was diminished and shifted negatively, and a new redox couple appeared between the Fe(III)/Fe(II) and Fe(II)/Fe(I) redox couples. Interestingly, however, the new oxidation at ca. −0.17 V vs Fc/Fc^+^ and new reduction at ca. −2.6 V vs Fc/Fc^+^ observed in the presence of NBu_4_^+^ are not observed in KPF_6_. This suggests these redox events were tied to the NBu_4_^+^-induced degradation, while the other degradation features could be caused by some interactions with KCl or Cl^–^ formed after the Cl^–^ is abstracted from Fe(III)TPP-Cl after the reduction from Fe(III) to Fe(II). This is supported by the CVs collected of FeTPP-PF_6_ before and after charge/discharge cycling (Figure 5, bottom), which do not show the development of the redox couple between Fe(III)/Fe(II) and Fe(II)/Fe(I). The only observed change is a slight negative shift of the Fe(III)/Fe(II) couple.

**Figure 6:**
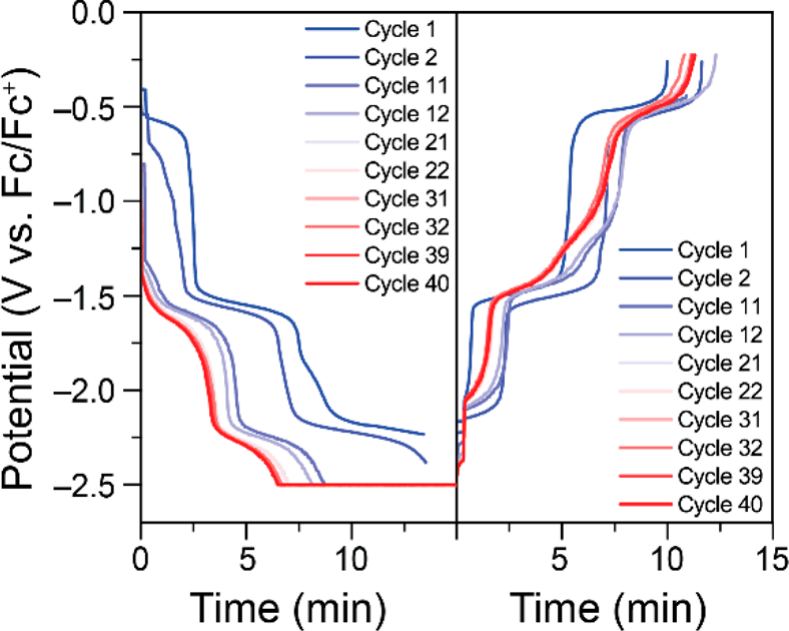
Selected representative potential vs time curves for 0.69 mM Fe(III)TPP-PF_6_ in 0.1 M KPF_6_ in DMF during charge (left) and discharge (right) cycling.

**Figure 7:**
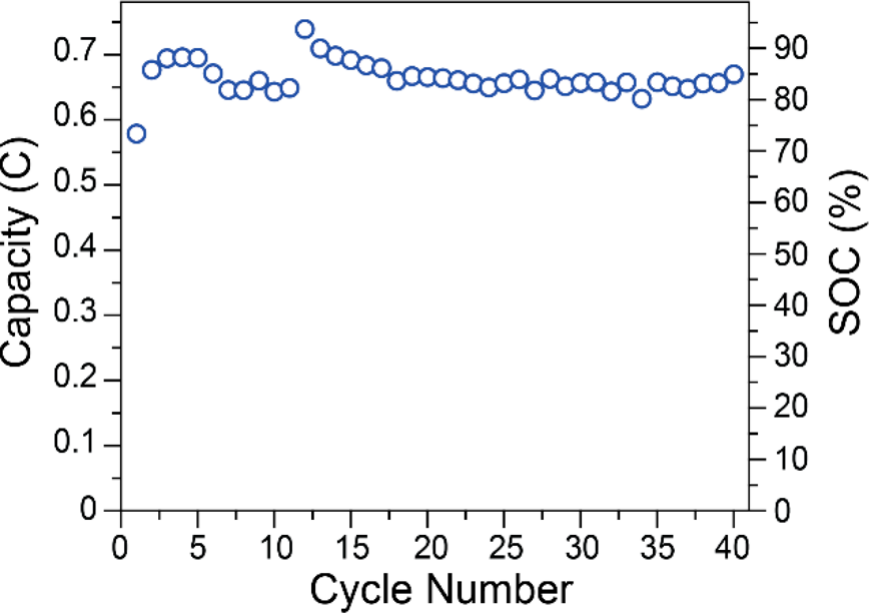
Discharge capacities and % SOC for charge/discharge cycling of 0.69 mM Fe(III)TPP-PF_6_ in 0.1 M KPF_6_ in DMF.

Fe(III)TPP-PF_6_ was tested in longer charge/discharge cycling in 0.1 M KPF_6_ in DMF at ±1.0 mA (Figures 6, 7, and S22–S23). The discharge capacity remains relatively stable over the 40 cycles, with a drop of 0.31% per cycle from the maximum observed (94% SOC in cycle 12) to 85% SOC in cycle 40.

CVs collected after the 10th and 40th charge/discharge cycles (Figure S24) show that some level of degradation is occurring even in these conditions though to a significantly lesser extent than when using FeTPP-Cl in NBu_4_PF_6_. The *E*_1/2_ values of the redox couples for Fe(II)/Fe(I) and Fe(I)/Fe(0) are unchanged, but peak currents for their respective reductions are diminished by 38 and 37% of their original values, respectively, which is greater than the observed capacity fade. This may be due to the appearance of a new redox couple at −1.29 V vs Fc/Fc^+^, which can be seen in the CV collected after the 10th cycle and remained for the duration of charge/discharge cycling. The Fe(III)/Fe(II) redox couple is the most affected of the three expected redox couples. The degradation products appear stable on the time scale of the electrochemical experiments.

**Figure 8:**
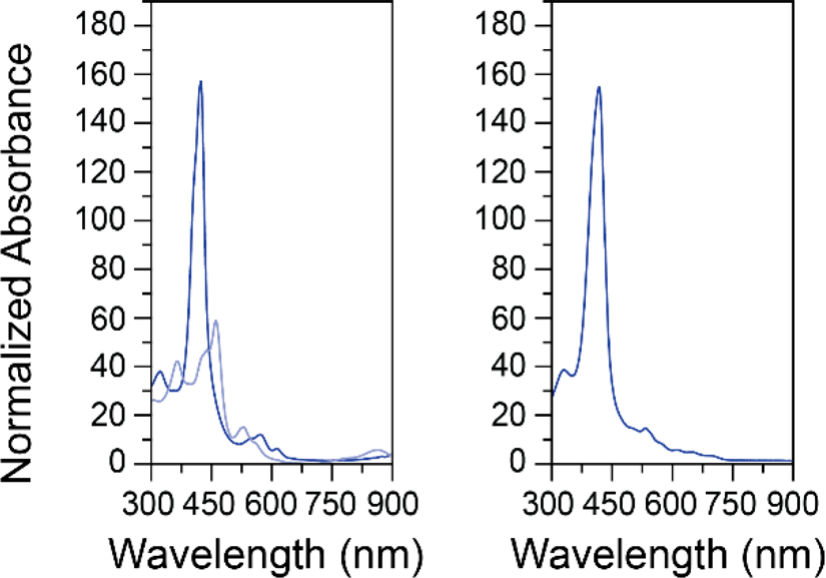
UV–vis absorption spectra of Fe(III)TPP-PF_6_ in 0.1 M KPF_6_ in DMF. Left: in a spectro-electrochemical cell (0.10 mM with 1.7 mm path length) at no applied potential (dark blue) and at −2.50 V (light blue). Right: in a cuvette (0.014 mM with 1 cm path length) after 40 charge–discharge cycles. Absorbance is normalized to the path length and to the concentration.

UV–vis spectra of the anolyte solution of Fe(III)TPP-PF_6_ in 0.1 M KPF_6_ in DMF was collected after charge/discharge cycling and showed the expected intensity and general features for Fe(III)TPP after reoxidation (Figure 8, right).^68^ Indeed, the absorbance broadly matches what is observed in a spectro-electrochemical cell (Figure 8, left) at the start of the experiment. After a reducing potential is applied, the absorbance profile evolves as the FeTPP-PF_6_ is reduced (Figure 8, left, light blue). A similar reduced porphyrin absorbance profile is obtained when the spectro-electrochemistry is repeated starting with FeTPP-Cl in 0.1 M KPF_6_ in DMF (Figure S25). This is expected as [Fe(0)TPP]^2–^ is expected to be generated in both conditions when the potential is held at −2.50 V vs Fc/Fc^+^, well negative of the reduction potential for [Fe(I)TPP]^−^. The spectra for Fe(III)TPP-Cl were mostly reformed after bulk reoxidation to Fe(III)TPP (Figure S25),^68,69^ however at a lower intensity and with the addition of a broad feature at 935 nm attributed to KCl, which was not observed in the case of FeTPP-PF_6_.^70^

Overall, from the charge/discharge cycling and capacity profiles, the stability of the Fe(III)TPP-PF_6_ in KPF_6_ system is enhanced compared to that of Fe(III)TPP-Cl in NBu_4_PF_6_. While the discharge capacity appears to be more stable for FeTPP-Cl in Figure 4, the CVs in Figure 5 highlight that most of the discharge capacity recorded does not originate from FeTPP-Cl as such but from unspecified degradation products generated as soon as the first cycle. Further cycling leads to further degradation, with a diminution of the current for the reduction of Fe(I) to Fe(0) by over 63% of its original value after 57 cycles (Figure S15). Conversely, while Fe(III)TPP-PF_6_ saw an apparently larger loss in discharge capacity during the first 40 charge/discharge cycles in KPF_6_ in DMF, CVs collected before and after show less degradation. The degradation products appear more stable to charge/discharge cycling than the putative alkyl iron complexes formed with FeTPP-Cl in NBu_4_PF_6_ in DMF. This is corroborated by the UV–vis data recorded after charge/discharge cycling and in spectro-electrochemistry experiments (Figures 8 and S25).

## Conclusion

The salt used as a support electrolyte, despite the common perception that it is chemically inert, is vitally important in redox flow battery systems. Herein, we have reported the electrochemical properties and charge/discharge cycling of Fe(III)TPP-Cl and Fe(III)TPP-PF_6_ in DMF with NBu_4_PF_6_ and KPF_6_ as support electrolytes. Fe(III)TPP-Cl, which is regularly used in the presence of NBu_4_PF_6_ for electrocatalysis of CO_2_ reduction, oxygen reduction, and the hydrogen evolution reaction, cannot be directly adapted to a RFB system using common supporting electrolytes due to the reactivity of the Fe(0) with tetraalkylammonium cations. This work serves to highlight the possible parasitic reactions that can occur with alkylammonium salts in nonaqueous solvents.

Fe(III)TPP-Cl was shown to still undergo degradation reactions when charged and discharged in KPF_6_ in DMF despite there being no apparent degradation product in the UV–vis absorbance spectra except for KCl. Exchanging Cl^–^ for PF_6_^–^ resulted in a system showing stable charge/discharge cycling for 40 cycles with a capacity fade of less than 0.5% per hour. To implement iron tetraphenylporphyrins as possible candidates for redox flow battery systems, the mechanism of degradation and nature of products formed require more studies to further diminish the possibility of deleterious side reactions. These considerations warrant a more careful look into the evaluation of common molecular electrocatalysts for use as an anolyte or catholyte in redox flow battery systems. A breadth of knowledge already exists as to expected electrochemical properties, and synthetic methods are available to tune both the redox potentials and solubility of molecular complexes traditionally used in electrocatalytic reactions. This could fast track their assessment as RFB anolytes or catholytes.

## Supporting Information Available

Supplemental data and analysis (UV–vis spectroscopy and electrochemical data) as referenced in the text (PDF)
