# Supplementary material for: Investigation of Iron(III) Tetraphenylporphyrin as a Redox Flow Battery Anolyte: Unexpected Side Reactivity with the Electrolyte
Source: J Phys Chem C Nanomater Interfaces. 2023 Jun 1;127(23):10938–46. doi: 10.1021/acs.jpcc.3c01763 (PMC10278133; doi:10.1021/acs.jpcc.3c01763)
Supplement: Supplementary file 1 — jp3c01763_si_001.pdf [file jp3c01763_si_001.pdf]

Supporting Information

Investigation of Iron(III) Tetraphenylporphyrin as a Redox Flow Battery Anolyte: Unexpected Side Reactivity with the Electrolyte

*Nathan H. Mitchell, Noémie Elgrishi**

Louisiana State University, Baton Rouge, LA, 70803, USA

*Correspondence to: noemie@lsu.edu

Index		*Page*
**Custom H-Cell Used for Charge/Discharge Cycling**	Fig. S1	SI-3
**Solubility Determination (UV-Vis spectra)**		
For Fe(III)TPP-Cl in DMF	Fig. S2	SI-4
For Fe(III)TPP-PF_6 in DMF	Fig. S3	SI-4
**Methodology Used to Determine D_0 and k_0 Values**		SI-5
**Electrochemical Characterization in 0.1 M NBu_4PF_6 in DMF**		
For Fe(III)TPP-Cl	Fig. S4-S8	SI-6,7,8
For Fe(III)TPP-PF_6	Fig. S9-S12	SI-9&10
**Charge/Discharge Cycling in 0.1 M NBu_4PF_6 in DMF**		
For Fe(III)TPP-Cl, long term charge/discharge	Fig. S13-S14	SI-11
For Fe(III)TPP-Cl, CVs after cycling	Fig. S15	SI-12
For Fe(III)TPP-PF_6, first charge/discharge cycle	Fig. S16	SI-12
**Electrochemical Characterization in 0.1 M KPF_6 in DMF**		
For Fe(III)TPP-PF_6	Fig. S17-S20	SI-13&14
**Charge/Discharge Cycling in 0.1 M KPF_6 in DMF**		
For Fe(III)TPP-Cl, first charge/discharge cycle	Fig. S21	SI-15
For Fe(III)TPP-PF_6, long term charge/discharge	Fig. S22-S23	SI-15&16
For Fe(III)TPP-PF_6, CVs after cycling	Fig. S24	SI-16
**UV-Vis Spectro-electrochemistry in 0.1 M KPF_6 in DMF**		
For Fe(III)TPP-Cl	Fig. S25	SI-17
**References**		SI-17

Custom H-Cell Used for Charge/Discharge Cycling

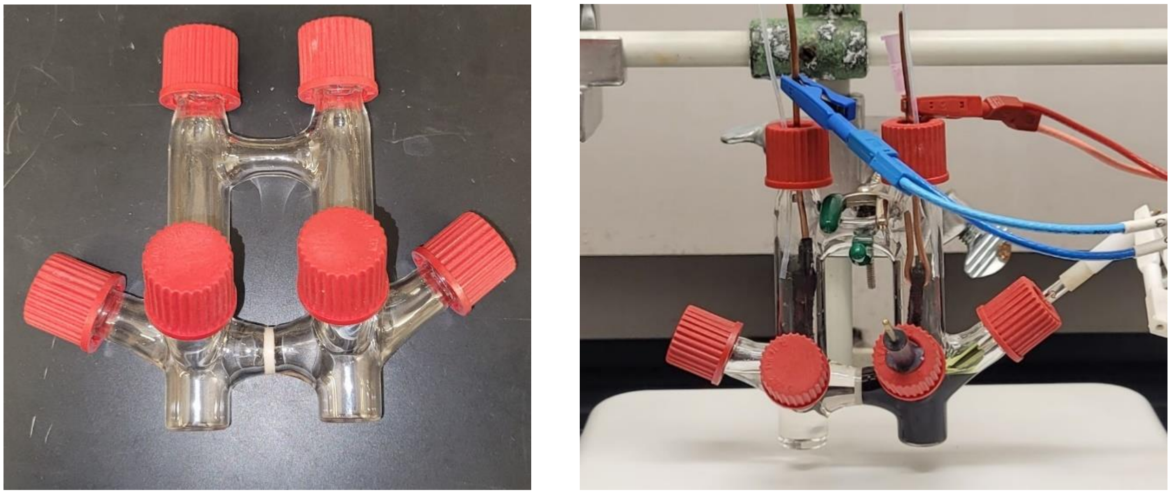

Figure S1. Custom H-Cell used for charge/discharge cycling. (Left) H-cell without solution present. (Right) H-cell loaded with 0.1 M NBu_4PF_6 in DMF on the left side and 0.5 mM Fe(III)TPP-Cl on the right side. The compartments are separated by a size P5 (ultrafine) porous glass frit.

Solubility Determination (UV-Vis spectra)

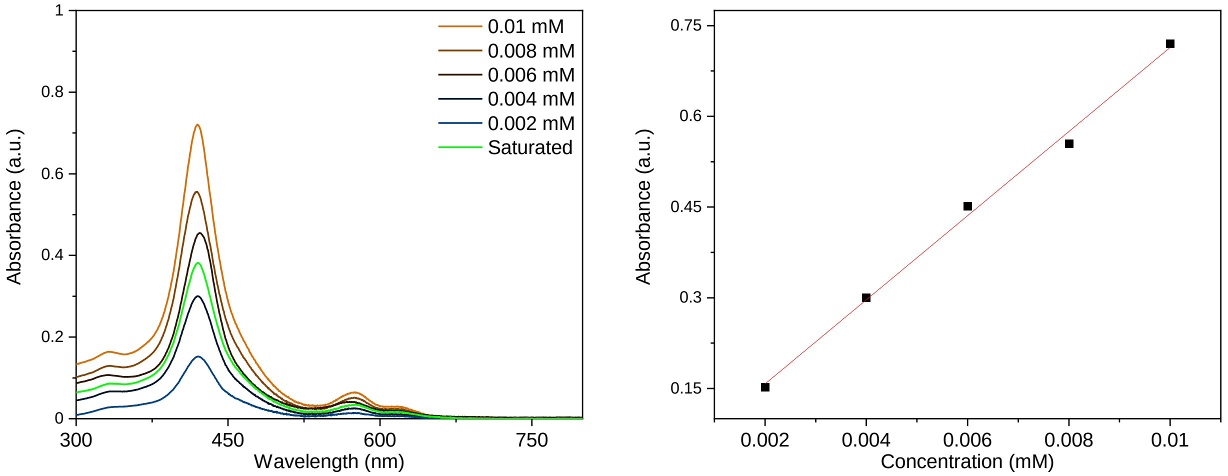

Figure S2. UV-Vis absorbance spectra of Fe(III)TPP-Cl in DMF used to determine the solubility of Fe(III)TPP-Cl. Left: Calibration curve UV-Vis absorbance spectra. The spectra from the saturated solution that was diluted into the calibration range is shown in green. Right: Calibration curve for Fe(III)TPP-Cl in DMF with the absorbance at 420 nm. Slope of the linear fit is 69.6±2.45 a.u. mM^{-1} (R^2=0.996)

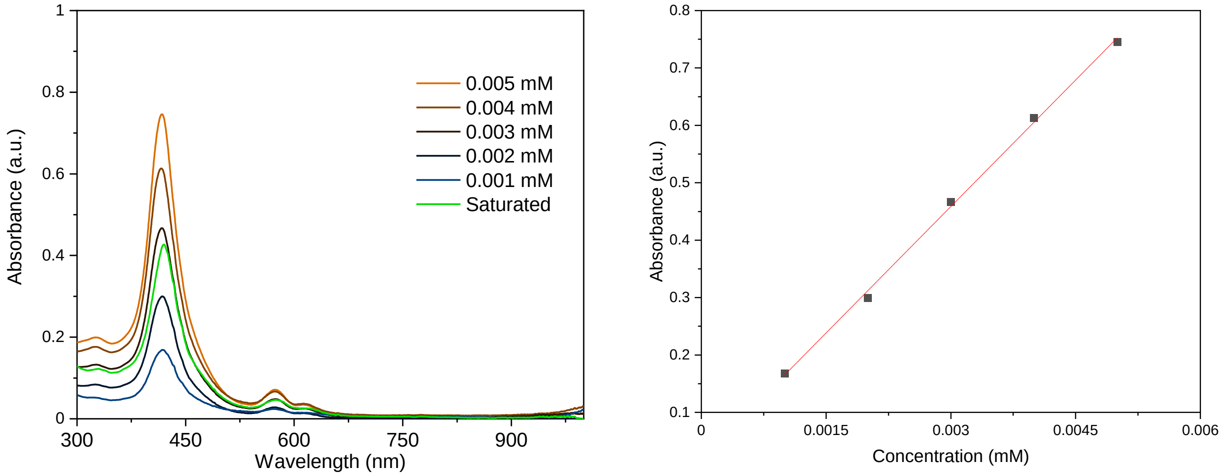

Figure S3. UV-Vis absorbance spectra of Fe(III)TPP-PF_6 in DMF used to determine the solubility of Fe(III)TPP-PF_6. Left: Calibration curve UV-Vis absorbance spectra. The spectra from the saturated solution that was diluted into the calibration range is shown in green. Right: Calibration curve for Fe(III)TPP-PF_6 in DMF with the absorbance at 417 nm. Slope of the linear fit is 146.9±3.32 a.u. mM^{-1} (R^2=0.998).

Methodology used to Determine D_0 and k_0 Values

Diffusion coefficients (D_0) were determined using Randles-Sevcik analysis. First the peak currents for each reduction and oxidation are determined and corrected for the baseline by using tangent lines. Peak currents (in A) are then plotted against the square root of scan rate (in V s^{-1}) and can then be fitted to the Randles-Sevcik equation (eq. S1):

$$i_p = 0.4463\left(\frac{F^3}{RT}\right)^{\frac{1}{2}} n^{\frac{3}{2}} A D_0^{\frac{1}{2}} C_0 \upsilon^{\frac{1}{2}} \qquad (S1)$$

Where i_p is peak current in *A*, *F* is Faraday's constant, *R* is the ideal gas constant in J mol^{-1} K^{-1}, *T* is temperature in K, *n* is the number of electrons in the redox reaction, *A* is the surface area of the electrode in cm^2, D_0 is the diffusion coefficient in cm^2 s^{-1}, C_0 is concentration in mol cm^{-3}, and υ is the scan rate in V s^{-1}.[1] The resulting slope of the line can be restated as equation S2

$$m = D_0^{\frac{1}{2}}\left(0.4463\left(\frac{F^3}{RT}\right)^{\frac{1}{2}} n^{\frac{3}{2}} A C_0\right) \qquad (S2)$$

Which can then be solved to find D_0.

Heterogeneous electron transfer rate constants (k_0) were determined using the method of Nicholson and Shain.[2–4] Using scan rate dependence data, peak-to-peak separations (in mV) are found for each redox couple. Peak-to-peak separations are then used to determine the Nicholson parameter (Ψ) for each scan rate using equation S3:

$$\Psi = \frac{-0.6288 + 0.0021\Delta E_p}{1 - 0.017\Delta E_p} \qquad (S3)$$

Where ΔE_p is peak-to-peak separation in mV.
After calculating the Nicholson parameter for each scan rate, they are plotted against the inverse square root of the scan rate (in V s^{-1}) using the following equation:

$$\Psi = k_0\left(\frac{\pi F \upsilon D}{RT}\right)^{-\frac{1}{2}} \qquad (S4)$$

Where k_0 is the electron transfer rate constant in cm s^{-1} D is the average of the diffusion coefficients for the reduced and oxidized species of each redox couple in cm^2 s^{-1}, F is Faraday's constant, υ is the scan rate in V s^{-1}, R is the ideal gas constant in J mol^{-1} K^{-1}, and T is temperature in K.[3,5] The slope of the resulting lines can be restated as equation S5:

$$\mathrm{m} = k_0\left(\frac{\pi F D}{RT}\right)^{-\frac{1}{2}} \qquad (S5)$$

Which can then be solved to find k_0.

Electrochemical Characterization in 0.1 M NBu_4PF_6 in DMF

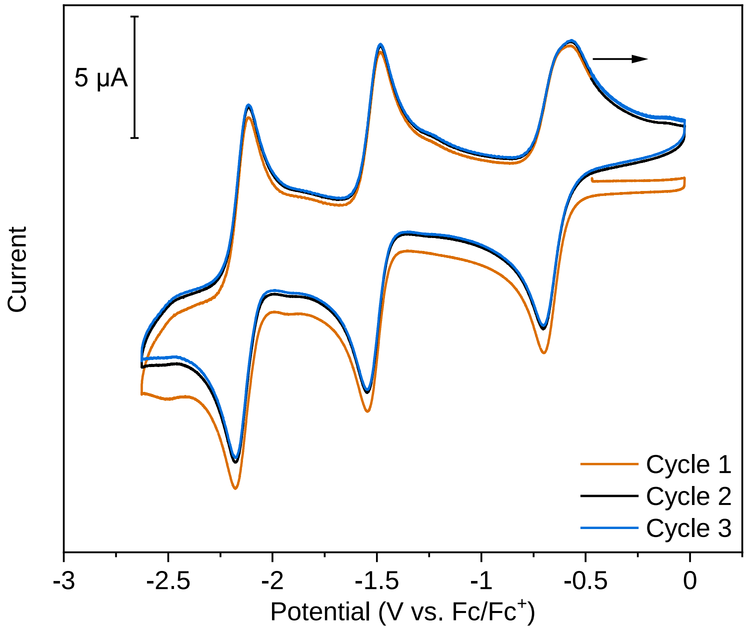

Figure S4. CV of 0.59 mM Fe(III)TPP-Cl in 0.1 M NBu_4PF_6 in DMF. CVs collected on glassy carbon working electrode at 100 mV/s. Three cycles are shown.

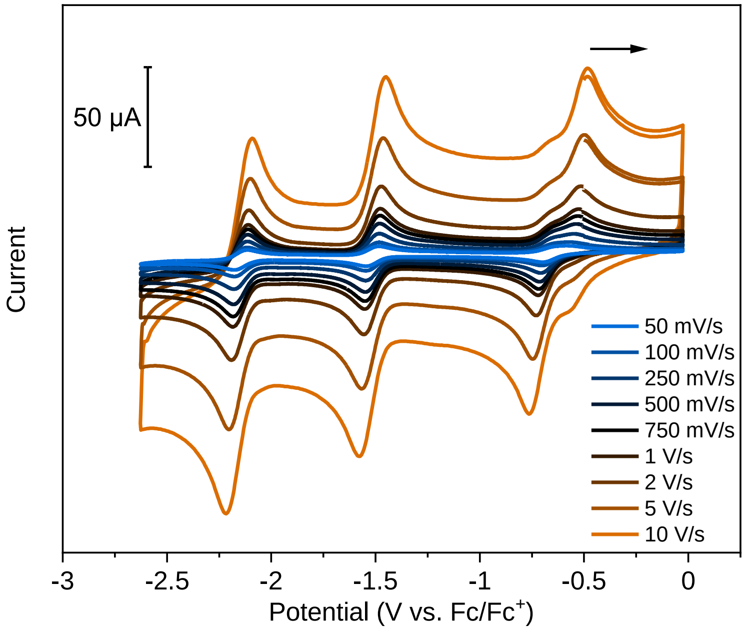

Figure S5. Scan rate dependence cyclic voltammetry of 0.59 mM Fe(III)TPP-Cl in 0.1 M NBu_4PF_6 in DMF. CVs collected on glassy carbon working electrode at various scan rates.

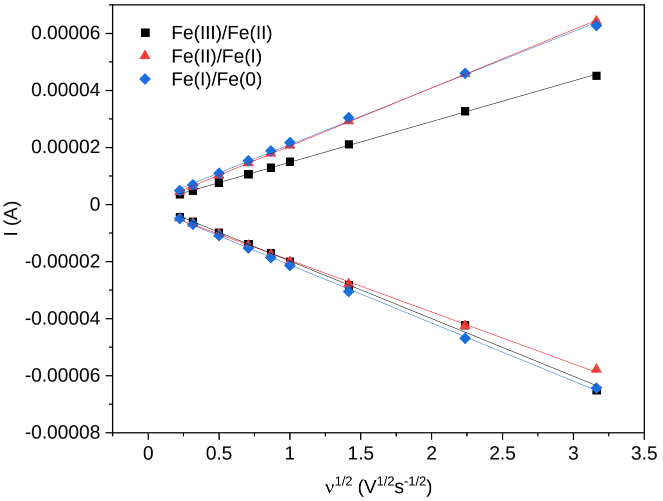

Figure S6. Randles-Sevcik Analysis of 0.59 mM Fe(III)TPP-Cl in 0.1 M NBu_4PF_6 in DMF. Current vs. square root of scan rate lines used for Randles-Sevcik analysis used to determine D_0. Slopes of the linear fits are as follows (units: A $V^{-1/2}$ $s^{1/2}$): Fe(II)/Fe(III) oxidation $1.43x10^{-5}$ $\pm 1.07x10^{-7}$ (R^2=1.000), Fe(I)/Fe(II) oxidation $2.04x10^{-5}$ $\pm 7.83x10^{-8}$ (R^2=1.000), Fe(0)/Fe(I) oxidation $1.98x10^{-5}$ $\pm 2.72x10^{-7}$ (R^2=0.998). Fe(III)/Fe(II) reduction $-2.02x10^{-5}$ $\pm 3.66x10^{-7}$ (R^2=0.997), Fe(II)/Fe(I) reduction $-1.82x10^{-5}$ $\pm 2.25x10^{-7}$ (R^2=0.999), Fe(I)/Fe(0) reduction $-2.04x10^{-5}$ $\pm 1.91x10^{-7}$ (R^2=0.999).

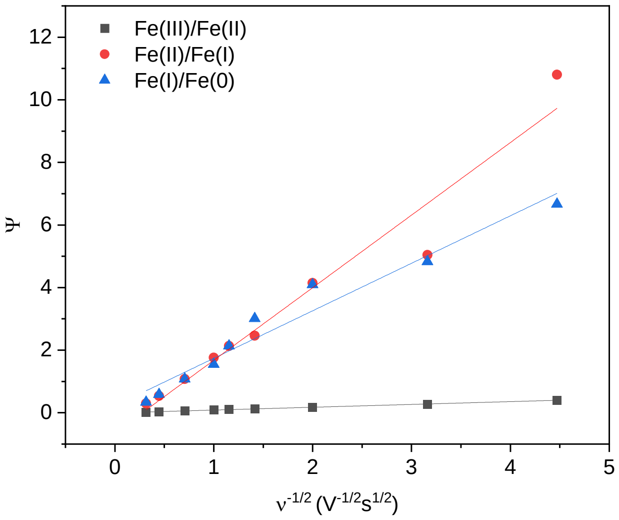

Figure S7. Nicholson method analysis of 0.59 mM Fe(III)TPP-Cl in 0.1 M NBu_4PF_6 in DMF. Nicholson parameter vs. inverse square root of scan rate lines used for the Nicholson method to determine k_0. Slopes of linear fits are as follows (units: $V^{1/2}$ $s^{-1/2}$): Fe(III)/Fe(II) 0.0901 ±0.0021 (R^2=0.996), Fe(II)/Fe(I) 2.32 ±0.19 (R^2=0.953), Fe(I)/Fe(0) 1.52 ±0.12 (R^2=0.956)

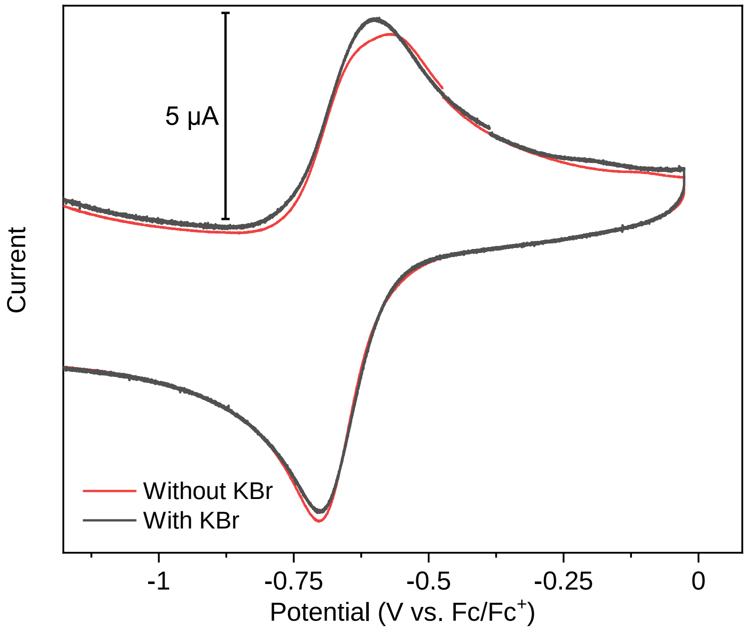

Figure S8. Cyclic voltammograms of 0.59 mM Fe(III)TPP-Cl with and without 20 equivalents of KBr in 0.1 M NBu_4PF_6 in DMF. CVs collected on glassy carbon at 100 mV/s.

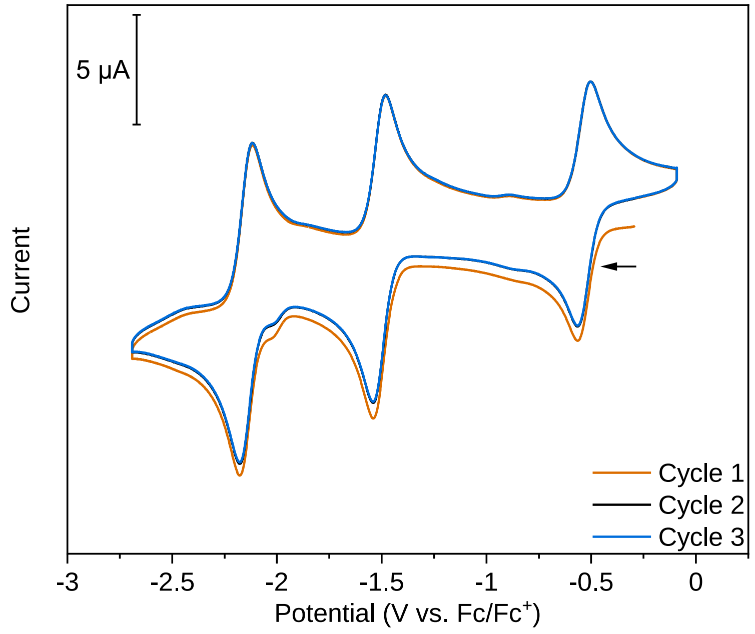

Figure S9. CV of 0.54 mM Fe(III)TPP-PF_6 in 0.1 M NBu_4PF_6 in DMF. CVs collected on glassy carbon working electrode at 100 mV/s. Three cycles are shown.

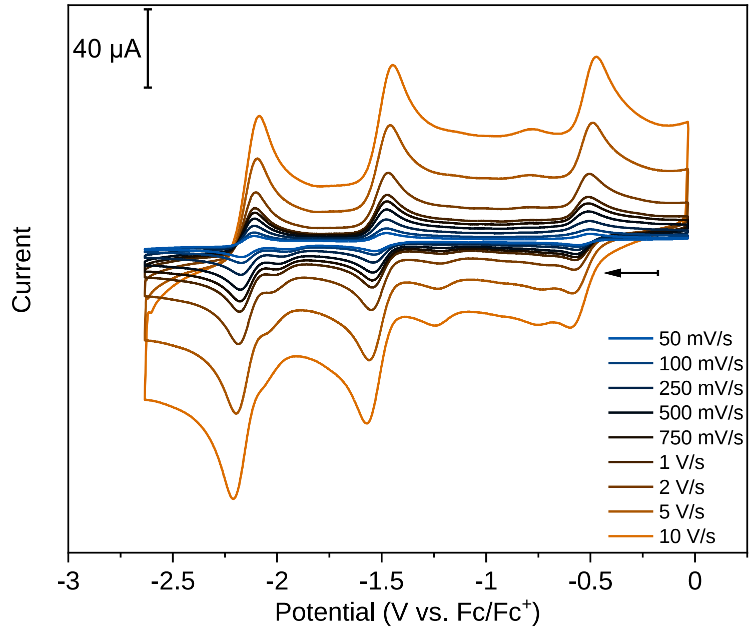

Figure S10. Scan rate dependence cyclic voltammetry of 0.54 mM Fe(III)TPP-PF_6 in 0.1 M NBu_4PF_6 in DMF. CVs collected on glassy carbon working electrode at various scan rates.

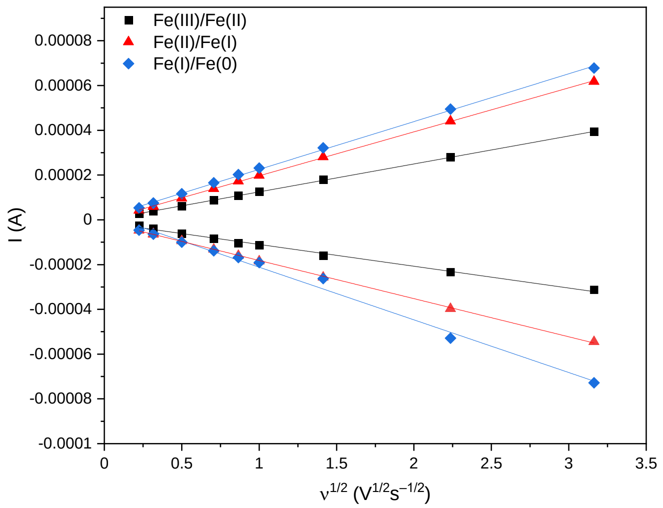

Figure S11. Randles-Sevcik Analysis of 0.54 mM Fe(III)TPP-PF_6 in 0.1 M NBu_4PF_6 in DMF. Current vs. square root of scan rate lines used for Randles-Sevcik analysis used to determine D_0. Slopes of the linear fits are as follows (units: A $V^{-1/2}$ $s^{1/2}$): Fe(II)/Fe(III) oxidation $1.25x10^{-5}$ ±$4.44x10^{-8}$ (R^2=1.000), Fe(I)/Fe(II) oxidation $1.97x10^{-5}$ ±$8.60x10^{-8}$ (R^2=1.000), Fe(0)/Fe(I) oxidation $2.14x10^{-5}$ ±$2.21x10^{-7}$ (R^2=0.999). Fe(III)/Fe(II) reduction $-9.75x10^{-6}$ ±$2.21x10^{-7}$ (R^2=0.996), Fe(II)/Fe(I) reduction $-1.71x10^{-5}$ ±$1.27x10^{-7}$ (R^2=1.000), Fe(I)/Fe(0) reduction $-2.35x10^{-5}$ ±$7.92x10^{-7}$ (R^2=0.991).

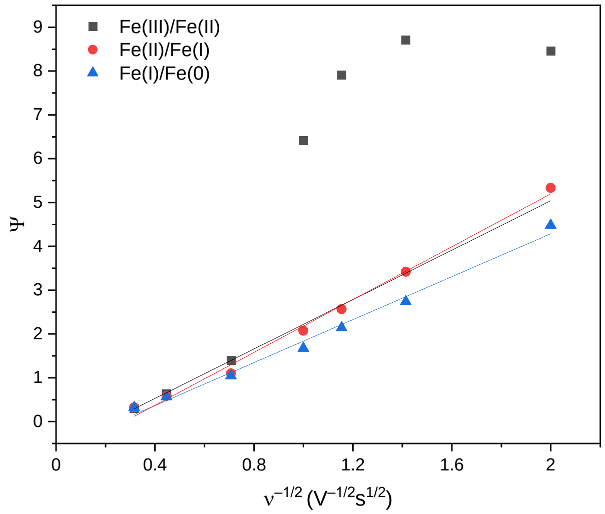

Figure S12. Nicholson method analysis of 0.54 mM Fe(III)TPP-PF_6 in 0.1 M NBu_4PF_6 in DMF. Nicholson parameter vs. inverse square root of scan rate lines used for the Nicholson method to determine k_0. Slopes of linear fits are as follows (units: $V^{1/2}$ $s^{-1/2}$): Fe(III)/Fe(II) 2.82 ±0.11 (R^2=0.999), Fe(II)/Fe(I) 3.02 ±0.11 (R^2=0.994), Fe(I)/Fe(0) 2.45 ±0.11 (R^2=0.991). The linear fit for the Fe(III)/Fe(II) only includes the data points at values less than 1 $V^{-1/2}$ $s^{1/2}$ as the peak-to-peak separation at lower scan rates for Fe(III)/Fe(II) was too small to be used in the Nicholson method as the resulting Nicholson parameters were outside of the working curve for the method.

Charge/Discharge Cycling in 0.1 M NBu_4PF_6 in DMF

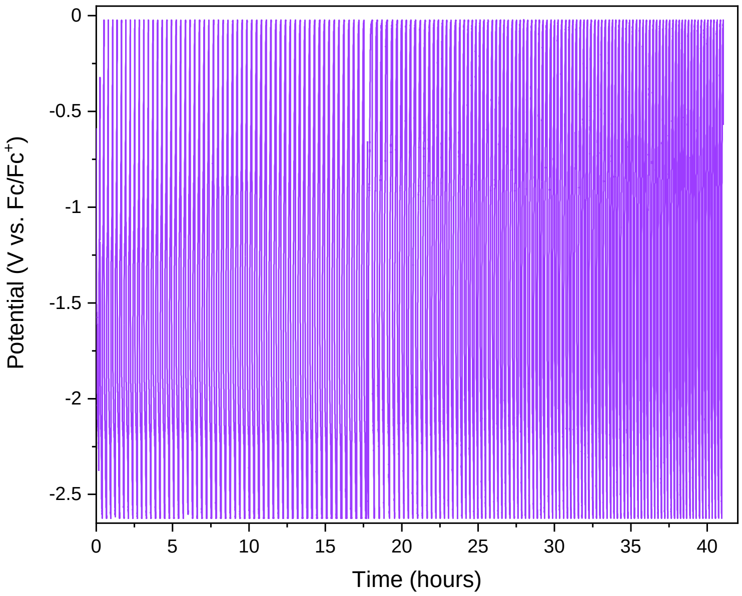

Figure S13. Charge/discharge cycling of 0.48 mM Fe(III)TPP-Cl in 0.1 M NBu_4PF_6 in DMF at ±1 mA. 151 cycles of galvanostatic cycling with potential limitation. During the first 57 cycles potential was held at the end of each charge for 20 minutes or until 100% SOC was reached. After the 57th cycle, charge was no longer held at 100% SOC. Data recorded on a reticulated vitreous carbon (RVC) working electrode.

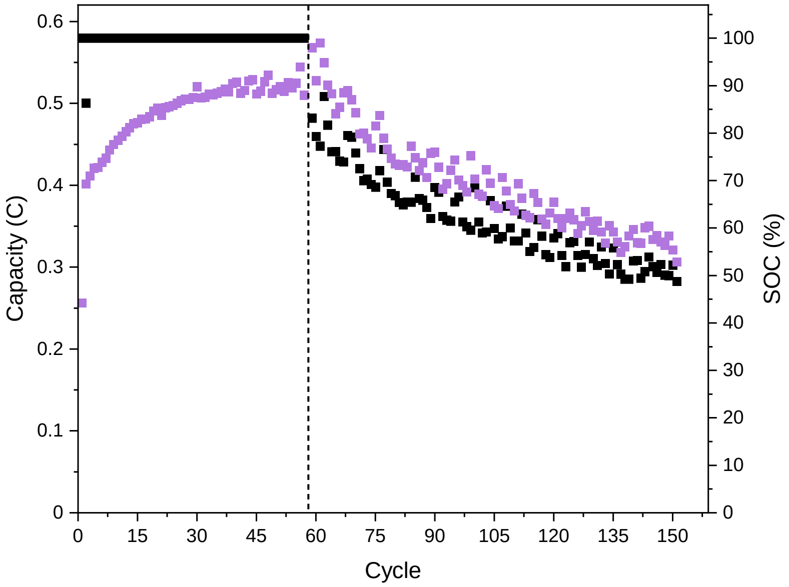

Figure S14. Charge (black) and discharge (purple) capacities and % SOC for charge/discharge cycling of 0.48 mM Fe(III)TPP-Cl in 0.1 M NBu_4PF_6 in DMF for 151 cycles. During the first 57 cycles potential was held at the end of each charge for 20 minutes or until 100% SOC was reached. After the 57th cycle (marked by the vertical dashed line), charge was no longer held at 100% SOC. Data recorded on an RVC working electrode.

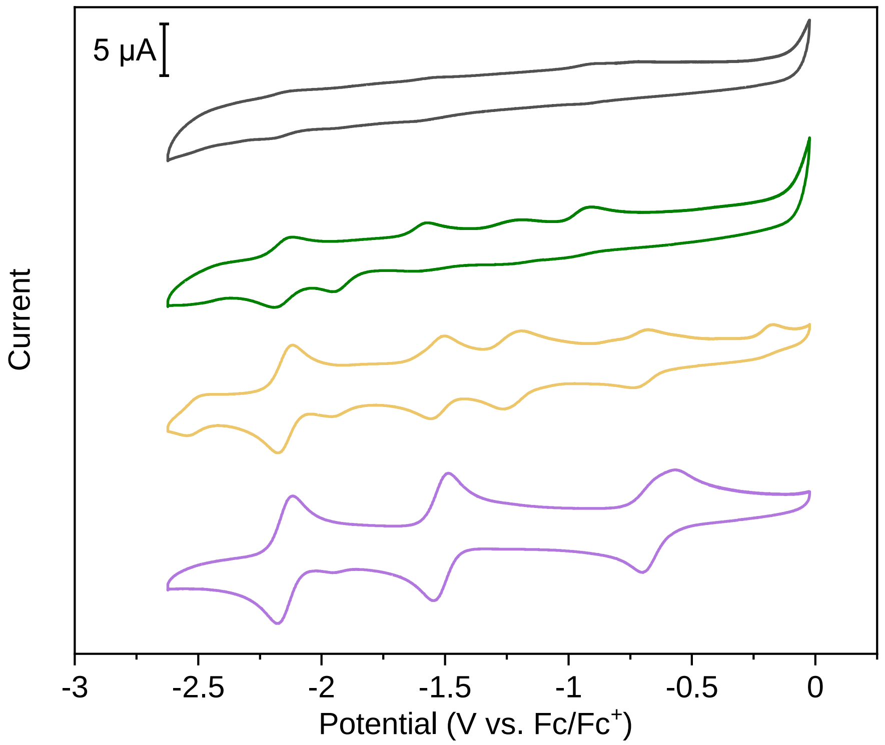

Figure S15 CVs of 0.48 mM Fe(III)TPP-Cl in 0.1 M NBu_4PF_6 in DMF collected on a glassy carbon working electrode at various points during charge/discharge cycling (from bottom to top). (Purple) Before charge/discharge cycling, (gold) after one charge/discharge cycle, (green) after 57 charge/discharge cycles, (black) after 151 charge/discharge cycles. Data recorded at 100 mV s^{-1}.

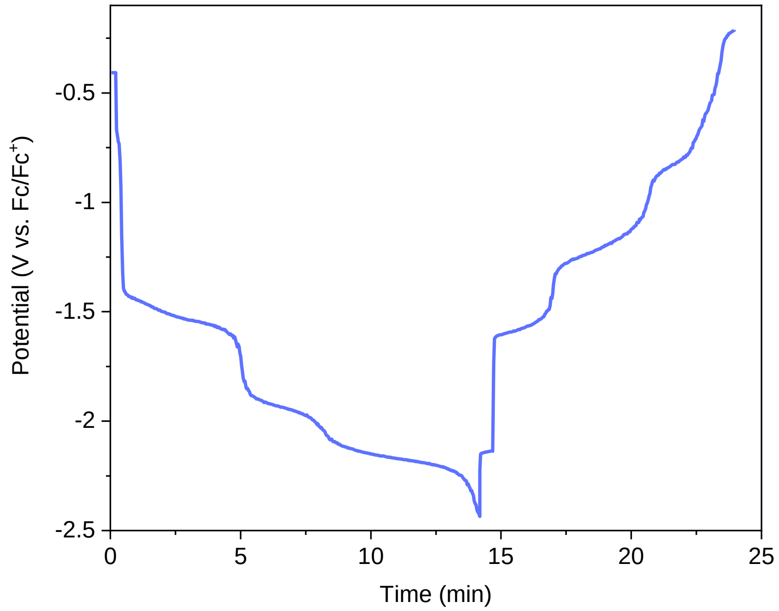

Figure S16. Single cycle charge/discharge cycling of 0.52 mM Fe(III)TPP-PF_6 in 0.1 M NBu_4PF_6 in DMF. Data recorded on an RVC working electrode.

Electrochemical Characterization in 0.1 M KPF_6 in DMF

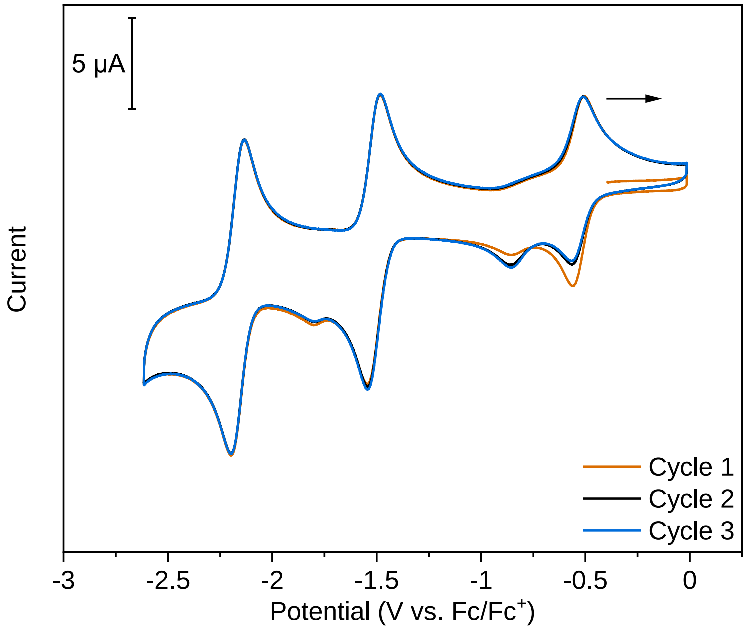

Figure S17. CV of 0.68 mM Fe(III)TPP-PF_6 in 0.1 M KPF_6 in DMF. CVs collected on glassy carbon working electrode at 100 mV/s. Three cycles are shown.

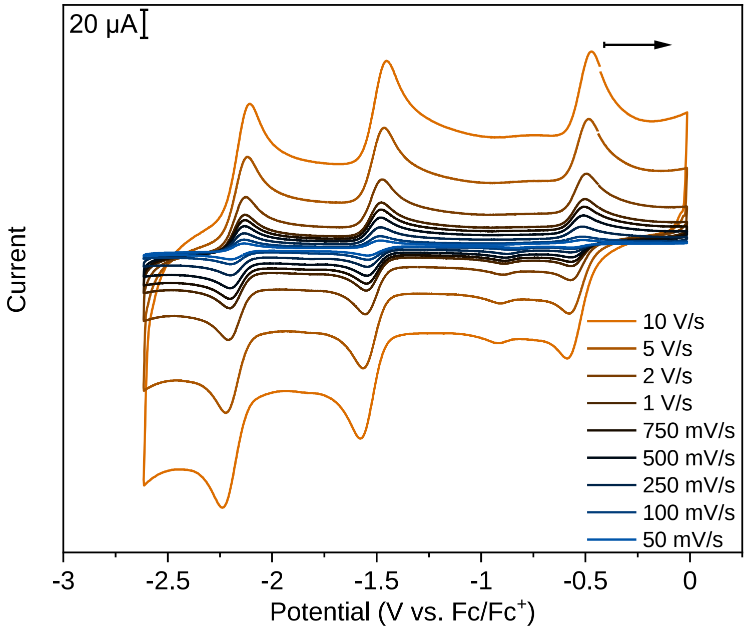

Figure S18. Scan rate dependence cyclic voltammetry of 0.68 mM Fe(III)TPP-PF_6 in 0.1 M KPF_6 in DMF. CVs collected on glassy carbon working electrode at various scan rates.

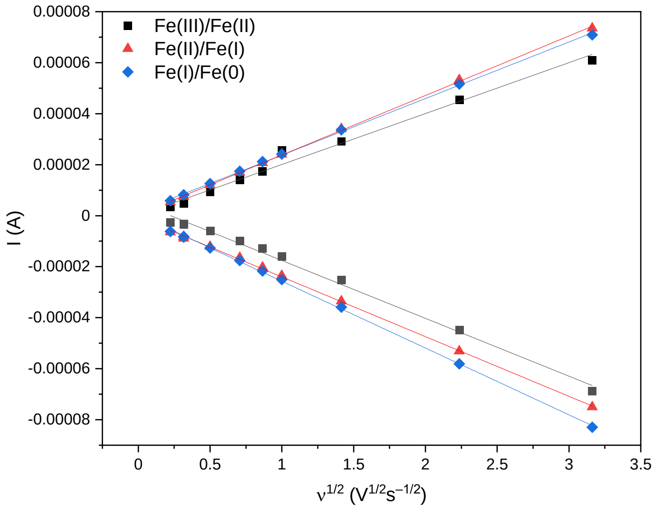

Figure S19. Randles-Sevcik Analysis of 0.68 mM Fe(III)TPP-PF_6 in 0.1 M KPF_6 in DMF. Current vs. square root of scan rate lines used for Randles-Sevcik analysis used to determine D_0. Slopes of the linear fits are as follows (units: A $V^{-1/2}$ $s^{1/2}$): Fe(II)/Fe(III) oxidation 2.00×10^{-5} $\pm8.23\times10^{-7}$ (R^2=0.987), Fe(I)/Fe(II) oxidation 2.34×10^{-5} $\pm1.60\times10^{-7}$ (R^2=1.000), Fe(0)/Fe(I) oxidation 2.22×10^{-5} $\pm1.99\times10^{-7}$ (R^2=0.999). Fe(III)/Fe(II) reduction -2.27×10^{-5} $\pm6.20\times10^{-7}$ (R^2=0.994), Fe(II)/Fe(I) reduction -2.34×10^{-5} $\pm2.01\times10^{-7}$ (R^2=0.999), Fe(I)/Fe(0) reduction -2.62×10^{-5} $\pm2.07\times10^{-7}$ (R^2=1.000).

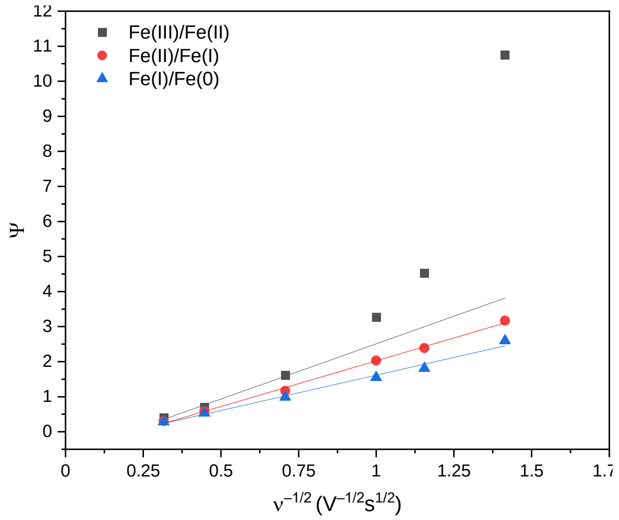

Figure S20. Nicholson method analysis of 0.68 mM Fe(III)TPP-PF_6 in 0.1 M KPF_6 in DMF. Nicholson parameter vs. inverse square root of scan rate lines used for the Nicholson method to determine k_0. Slopes of linear fits are as follows (units: $V^{1/2}$ $s^{-1/2}$): Fe(III)/Fe(II) 3.15 ±0.30 (R^2=0.991), Fe(II)/Fe(I) 2.61 ±0.074 (R^2=0.997), Fe(I)/Fe(0) 2.02 ±0.11 (R^2=0.988). The linear fit for the Fe(III/II) only includes the data points at values less than 1 $V^{-1/2}$ $s^{1/2}$ as the peak-to-peak separation at lower scan rates for Fe(III/II) was too small to be used in the Nicholson method as the resulting Nicholson parameters were outside of the working curve for the method.

Charge/Discharge Cycling in 0.1 M KPF_6 in DMF

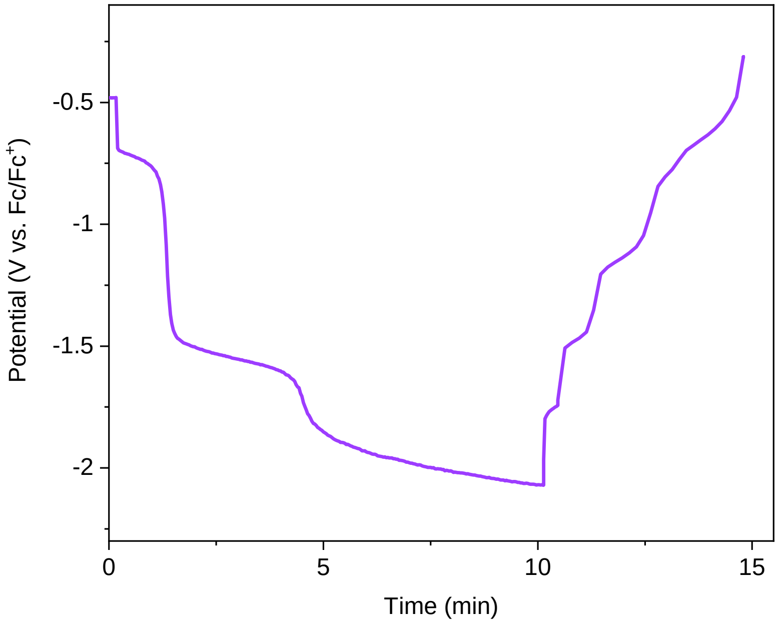

Figure S21. Single cycle charge/discharge cycling of 0.53 mM Fe(III)TPP-Cl in 0.1 M KPF_6 in DMF. Data recorded on an RVC working electrode.

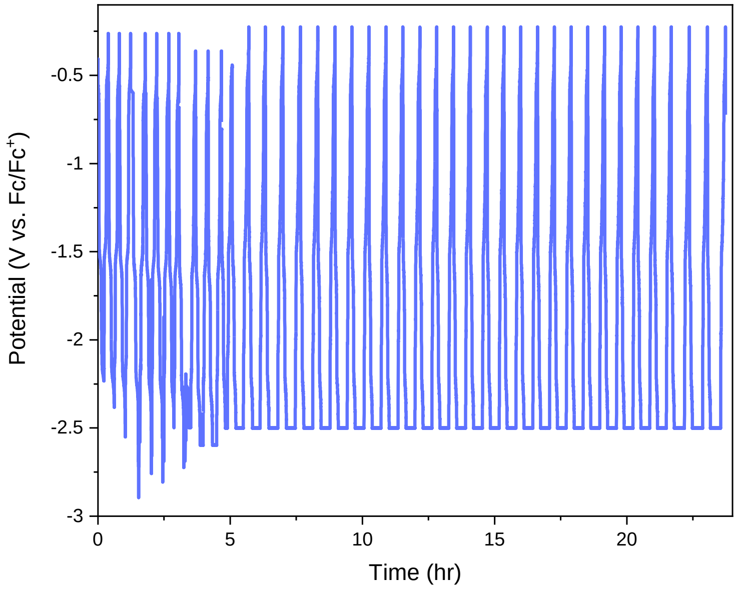

Figure S22. Charge/discharge cycling of 0.69 mM Fe(III)TPP-PF_6 in 0.1 M KPF_6 in DMF at ±1 mA. 40 cycles of galvanostatic cycling with potential limitation. Potential was held at the end of each charge for 20 minutes or until 100% SOC was reached. Data recorded on an RVC working electrode.

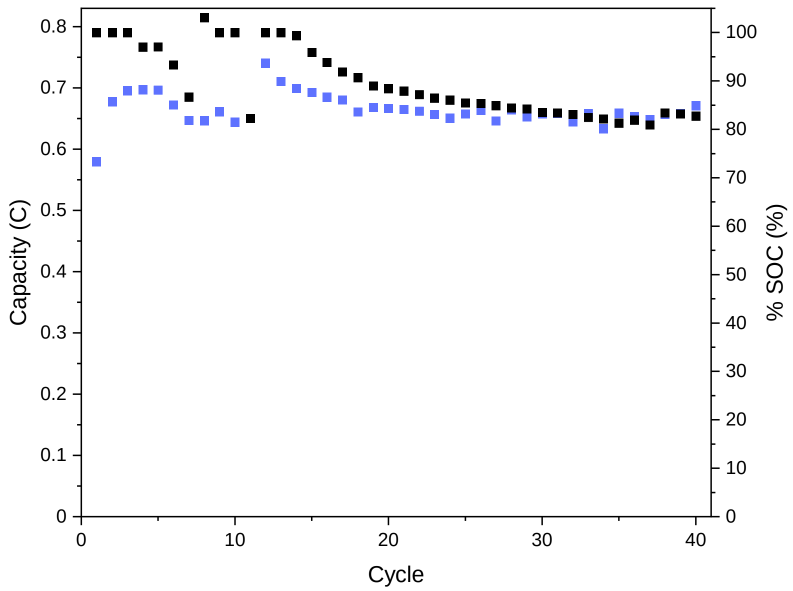

Figure S23. Charge (black) and discharge (blue) capacities and % SOC for charge/discharge cycling of 0.69 mM Fe(III)TPP-PF_6 in 0.1 M NBu_4PF_6 in DMF for 40 cycles. During charging, the potential limit was held for 20 minutes or until 100% SOC was reached. Data recorded on an RVC working electrode.

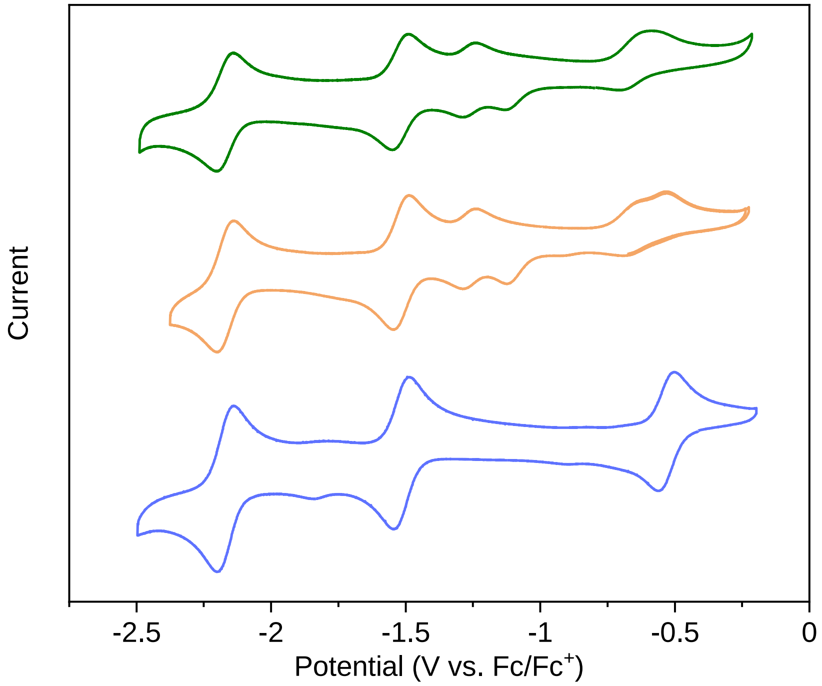

Figure S24. Cyclic voltammograms on glassy carbon electrodes of 0.69 mM Fe(III)TPP-PF_6 before (blue, bottom) charge/discharge cycling, after 10 charge/discharge cycles (orange, middle) and after 40 charge/discharge cycles (green, top) in 0.1 M KPF_6 in DMF. Data recorded at 100 mV s^{-1}.

UV-Vis Absorbance Spectro-electrochemistry in 0.1 M KPF_6 in DMF

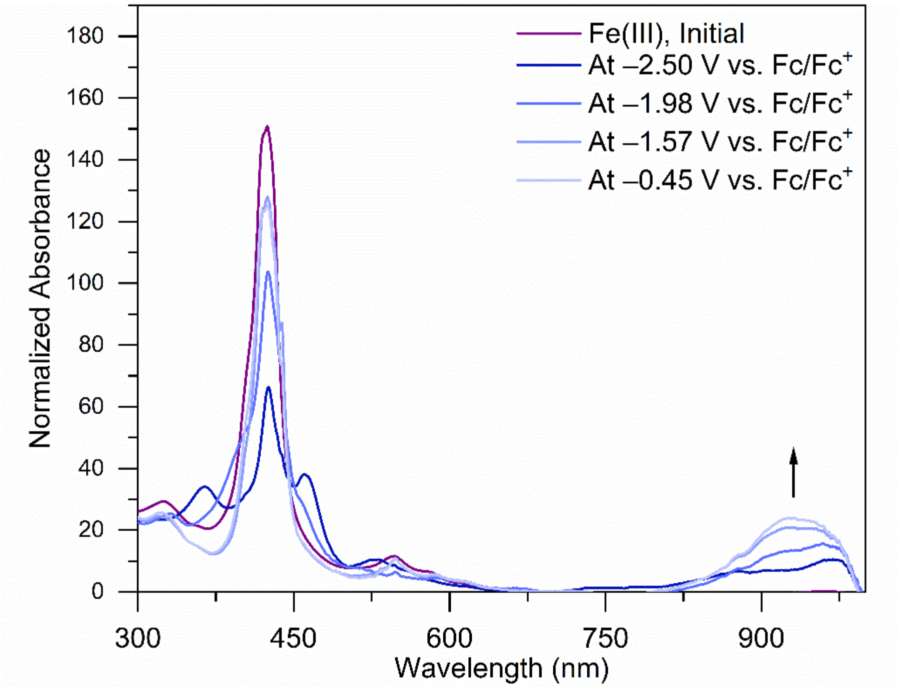

Figure S25. UV-Vis absorbance spectra of 0.1 mM Fe(III)TPP-Cl in 0.1 M KPF_6 in DMF in a spectro-electrochemical cell after chronoamperometry was performed at various applied potentials as specified on the graph.

References

(1) Elgrishi, N.; Rountree, K. J.; McCarthy, B. D.; Rountree, E. S.; Eisenhart, T. T.; Dempsey, J. L. A Practical Beginner's Guide to Cyclic Voltammetry. *J. Chem. Educ.* **2018**, *95* (2), 197–206. https://doi.org/10.1021/acs.jchemed.7b00361.

(2) Nicholson, R. S. Theory and Application of Cyclic Voltammetry for Measurement of Electrode Reaction Kinetics. *Anal. Chem.* **1965**, *37* (11), 1351–1355. https://doi.org/10.1021/ac60230a016.

(3) Wang, H.; Sayed, S. Y.; Luber, E. J.; Olsen, B. C.; Shirurkar, S. M.; Venkatakrishnan, S.; Tefashe, U. M.; Farquhar, A. K.; Smotkin, E. S.; McCreery, R. L.; Buriak, J. M. Redox Flow Batteries: How to Determine Electrochemical Kinetic Parameters. *ACS Nano* **2020**, *14* (3), 2575–2584. https://doi.org/10.1021/acsnano.0c01281.

(4) Lavagnini, I.; Antiochia, R.; Magno, F. An Extended Method for the Practical Evaluation of the Standard Rate Constant from Cyclic Voltammetric Data. *Electroanalysis* **2004**, *16* (6), 505–506. https://doi.org/10.1002/elan.200302851.

(5) Berben, L. A.; Arnold, A.; Dougherty, R. J.; Carr, C. R.; Reynolds, L. C.; Fettinger, J. C.; Augustin, A. A Stable Organo-Aluminum Analyte Enables Multielectron Storage for a Nonaqueous Redox Flow Battery. *J. Phys. Chem. Lett.* **2020**, *11* (19), 8202–8207. https://doi.org/10.1021/acs.jpclett.0c01761.
